# Occurrence of Metabolic Disorders in Bilateral Primary Aldosteronism Compared to Unilateral Primary Aldosteronism

**DOI:** 10.3390/diseases13020052

**Published:** 2025-02-10

**Authors:** Chiara Grasselli, Maicol Baldini, Lucia Salvi, Grazia Vestita, Maurizio Zizzo, Davide Felaco, Maria Carolina Balli, Giulia Besutti, Aurelio Negro, Angelo Ghirarduzzi

**Affiliations:** 1 Hypertension Unit of Second Internal Cardiovascular Medicine, Azienda Unità Sanitaria Locale-IRCCS di Reggio Emilia, 42123 Reggio Emilia, Italy; maicol.baldini@ausl.re.it (M.B.); lucia.salvi@ausl.re.it (L.S.); grazia.vestita@ausl.re.it (G.V.); aurelio.negro@ausl.re.it (A.N.); angelo.ghirarduzzi@ausl.re.it (A.G.); 2 Surgical Oncology Unit, Azienda Unità Sanitaria Locale-IRCCS di Reggio Emilia, 42123 Reggio Emilia, Italy; maurizio.zizzo@ausl.re.it; 3 Radiology Unit, Azienda Unità Sanitaria Locale-IRCCS di Reggio Emilia, 42123 Reggio Emilia, Italy; davide.felaco@ausl.re.it (D.F.); mariacarolina.balli@ausl.re.it (M.C.B.); giulia.besutti@ausl.re.it (G.B.); 4 Department of Medical and Surgical Sciences, University of Modena and Reggio Emilia, 41124 Modena, Italy

**Keywords:** metabolic syndrome, obesity, primary aldosteronism

## Abstract

Background: Metabolic syndrome (MetS) is a common comorbidity associated with hypertension that occurs more often in primary aldosteronism (PA). Our work aims to investigate the prevalence of MetS and its determinants in unilateral PA and bilateral PA, as confirmed by adrenal venous sampling (AVS). Methods: This was a retrospective, cross-sectional study. We investigated metabolic indicators in 160 cases of PA, categorized by AVS—82 with unilateral PA and 78 with bilateral PA. A control group of 80 non-PA patients with essential hypertension, matched for age and sex, was also included. Results: Unilateral PA had a higher aldosterone–renin ratio and lower serum potassium levels than bilateral PA. Nevertheless, bilateral PA exhibited a higher prevalence of MetS (41% vs. 30.5%; *p* = 0.001), obesity, BMI, LDL hypercholesterolemia, and hypertriglyceridemia than unilateral PA. Conclusions: Bilateral PA presents a greater incidence of MetS than unilateral PA, in spite of the latter showing a higher aldosterone–renin ratio and lower serum potassium levels. The results suggest that the mechanisms underlying MetS may differ between unilateral and bilateral PA.

## 1. Introduction

Primary aldosteronism (PA) encompasses a range of conditions characterized by excessive aldosterone excretion, which occurs despite a decrease in renin concentration [1]. This excessive aldosterone production primarily leads to hypertension and increased potassium loss, potentially resulting in hypokalemia [2]. PA is the predominant determinant of secondary hypertension, and the incidence of resistant hypertension in these patients ranges from 17% to 23% [3]. The recognition of primary hyperaldosteronism is crucial in the therapeutic approach to the patient in order to prevent the consequences associated with excess mineralocorticoids. Indeed, a higher incidence of myocardial infarction, stroke, and atrial fibrillation has been observed in patients with hyperaldosteronism compared to those with essential hypertension, despite similar characteristics in terms of the duration and severity of arterial hypertension [4].

Initially, it was hypothesized that aldosterone acts only on a limited number of epithelial target organs, such as the kidney, colon, and salivary glands, playing a central role in the control of the hydroelectrolytic balance [5]. Subsequently, it became clear that the spectrum of tissues and organs affected by the action of this mineralocorticoid hormone was broader than originally thought. In fact, aldosterone also acts on ‘non-epithelial’ target tissues such as the heart, blood vessels, and brain [6,7,8,9,10,11,12], leading to an increase in organ damage per se, independent of the increase in blood pressure caused by the excessive secretion of the hormone itself. It has been demonstrated that at the cardiac level, excessive aldosterone production induces fibrosis by directly stimulating cardiomyocytes and the proliferation of fibroblasts as an indirect reparative response to inflammation and cell death [13].

The high plasma concentrations of aldosterone are associated with an increased incidence and severity of left ventricular hypertrophy; in patients with essential hypertension and in patients with hyperaldosteronism, aldosterone levels correlate directly with the thickness of the left ventricular wall. In addition, an alteration in myocardial structure has been noted, with an increase in the fibrotic component and a reduction in ventricular wall compliance [14,15,16,17]. Aldosterone acts on blood vessels at the endothelial level by decreasing the bioactivity of nitric oxide (NO); one hypothesis is that aldosterone, similarly to angiotensin II, induces endothelial oxidative stress, leading to the degradation of NO [18]. At the wall level, aldosterone reduces vascular compliance by decreasing the distensibility of the smooth muscle [19]. Finally, aldosterone increases the levels of PAI-1 (plasminogen activator inhibitor-1), with a prothrombotic and antifibrinolytic effect [20]. At the encephalic level, high plasma levels of aldosterone are considered a risk factor for the onset of stroke, as demonstrated in experiments on SHRSP rats (stroke-prone spontaneously hypertensive rats) [21]. It is possible that the reduced diameter of blood vessels in SHRSP rats is the result of vascular remodeling, at least in part due to the increased proliferation of smooth muscle cells, probably because of their increased responsiveness to EGF (epidermal growth factor) [22]. Another study suggested that the EGF signaling pathway may be altered in mineralocorticoid-induced hypertension [23].

In addition to the effects on the aforementioned organs, a bidirectional relationship between aldosterone and insulin resistance has recently emerged, which is the pathogenic basis of metabolic syndrome.

**Figure 1:**
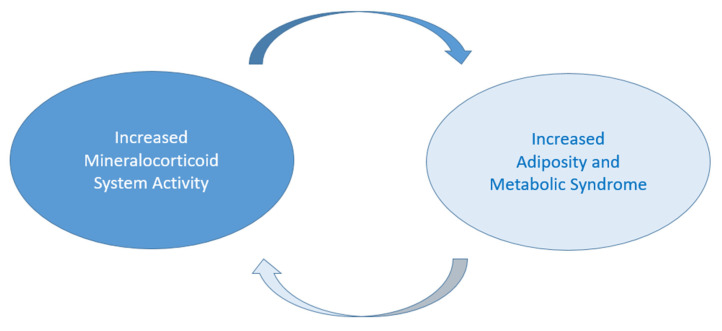
Relationship between mineralocorticoid (MC) system activity and metabolic syndrome (MetS). Increased MC activity can lead to MetS and adiposity, which may, in turn, contribute to the maintenance of MC activity, resulting in the further progression of adiposity and MetS.

Metabolic syndrome (MetS) refers to a multifaceted condition that includes obesity, hyperglycemia, dyslipidemia, and hypertension, and is associated with an increased likelihood of insulin resistance and heart-related conditions [24]. Both metabolic syndrome [25] and PA [26] are associated with increased inflammatory burden. Therefore, a relationship between the two is possible. Indeed, excess aldosterone could contribute to the maturation and dysfunction of adipocytes by binding to mineralocorticoid receptors on adipose tissue, leading to an imbalance of adipokines; conversely, these increasing pro-inflammation adipokines would directly act on the adrenal glands producing aldosterone, causing metabolic disorders. In particular, human adipocytes can indeed secrete MC-releasing factors [27] and various adipokines such as leptin, which can stimulate the adrenal production of aldosterone [28,29,30]. In addition, adipose tissue can produce angiotensinogen and possesses a local renin–angiotensin system (RAS) [31]. It has been reported that plasma and adipose tissue’s MC activity is significantly reduced after successful weight loss by calorie restriction [32,33]. Together, these findings support the concept of increased MC activity as a mediator of MetS and obesity, which, in turn, contributes to the maintenance of high MC activity, resulting in the progression of these conditions (Figure 1).

Previous research has shown that hyperaldosteronism and hypokalemia, which are common in PA, are strongly associated with biochemical imbalance [34,35,36]. PA may involve only one adrenal gland, typically attributable to aldosterone-producing adenoma (APA), or it may affect both adrenal glands, usually in cases of bilateral adrenal hyperplasia (BAH). These subtypes differ in their aspects and underlying etiology [37]. APA is characterized by higher levels of aldosterone and lower levels of potassium [38], and this could theoretically account for the higher incidence of MetS compared to BAH.

In contrast, a previous study involving 50 patients with APA and 50 with BAH found that the incidence of metabolic syndrome was significantly higher in those with bilateral PA compared to those with APA (62% vs. 36%; *p* < 0.05) [39]. Another study corroborated these findings, showing a greater prevalence of MetS in bilateral PA in contrast to APA (79.8% vs. 64.7%; *p* < 0.05) [40]. Additionally, a recent extensive study revealed that obesity was more prevalent among patients with BAH than in those with APA [40], implying that the two forms of PA have different incidences of MetS, regardless of aldosterone and potassium levels.

Thus, the aim of our study was to compare the incidence of MetS in the two forms of PA identified by adrenal venous sampling (AVS) and to determine whether there are common characteristics among patients who presented with MetS.

## 2. Materials and Procedures

### 2.1. Patients

Between January 2014 and May 2024, 160 hypertensive patients diagnosed with primary aldosteronism (PA), according to a standardized diagnostic protocol at our center, who were classified into subtypes via AVS participated in the study. In addition, 80 hypertensive patients with essential hypertension (EH), matched for age (±5 years) and sex, were included as a control cohort.

The presence of other secondary causes of hypertension, such as Cushing’s syndrome, pheochromocytoma, renal artery stenosis, or nephroparenchymal hypertension, was an exclusion criterion for the study.

Prior to screening, all participants were instructed to discontinue drugs that could potentially interfere, such as angiotensin-converting enzyme inhibitors, angiotensin receptor blockers, dihydropyridines, and β-blockers for a minimum of 4 weeks; diuretics and antialdosteronics for a minimum of 6 weeks; oral contraceptives in women of childbearing age; and hormone replacement therapy in menopausal women. To control blood pressure values, we used drugs with minimal effects on plasma aldosterone concentration (PAC), plasma renin activity (PRA), and plasma active renin (DRC) levels, such as calcium antagonists or α1-blockers, either alone or in combination. To normalize potassium concentrations, we administered oral potassium supplements [41]. The patients were all residents of the same region and had similar dietary habits. Specific diets related to ethnic background or religious beliefs were suspended for the entire study period. No patient was treated with hypolipidemic drugs throughout the study period. None of the patients in the study participated in competitive sports.

All participants provided informed consent.

### 2.2. PA Recognition

As per the 2020 SIIA guidelines for the approach to PA [42], the initial screening of patients was performed using the aldosterone-to-renin ratio (ARR). The ARR was considered positive when greater than 30 ng/dL·ng·mL^−1^·h^−1^ or 4000 pmol/liter·ng·L^−1^·s^−1^, in conjunction with an aldosterone level greater than 10 ng/dL (416 pmol/L). Blood samples were collected while the patient was seated, between 08:00 and 10:00.

The confirmatory test consisted of a 2 L intravenous saline load (0.9% NaCl injected over a 4 h period). If aldosterone levels were greater than 5 ng/dL (138.7 pmol/L), the result was considered positive [43]. The minimum thresholds for plasma renin activity (PRA) and active plasma renin concentration (DRC) were 0.1 ng·mL/h and 0.4 ng/L, respectively.

### 2.3. Subtype Diagnosis

The type of PA was determined using AVS [44], conducted by a team of experienced radiologists (G.G., D.F., and M.C.B.) who had also previously reviewed the patients’ CT or MRI scans. The success of AVS was determined when the adrenal vein/inferior vena cava cortisol gradient was 2 or more (selectivity index—SI). Significant lateralization was detected when the aldosterone/cortisol (A/C) ratio in one adrenal gland was more than four times higher than in the contralateral gland (lateralization index—LI), or when it was three times higher in the ipsilateral gland than in the contralateral gland, together with a lower A/C ratio in the contralateral gland than in the peripheral vein (contralateral ratio—CLR) [43].

Finally, all patients were screened for glucocorticoid-remediable aldosteronism (GRA) using long polymerase chain reaction (PCR) techniques to exclude the presence of the chimeric CYP11B1/CYP11B2 gene [45,46].

### 2.4. Definition of MetS

MetS was defined according to the 2001 National Cholesterol Education Program-Adult Treatment Panel III (NCEP-ATPIII) criteria [47]. A diagnosis of MetS depends on the presence of at least three of the following conditions: increased waist circumference (≥102 cm for men and ≥88 cm for women), elevated triglyceride levels (≥150 mg/dL or current use of triglyceride-lowering medication), low HDL cholesterol levels (<40 mg/dL for men and <50 mg/dL for women), elevated blood pressure (≥130/85 mmHg or current use of antihypertensive treatment), and fasting plasma glucose ≥100 mg/dL (or current use of hypoglycemic agents).

### 2.5. Measures

We collected data on age, sex, body mass index (BMI), systolic and diastolic blood pressure (SBP and DBP), and laboratory measurements of circulating potassium, sodium, PAC, PRA, DRC, fasting blood glucose, total cholesterol (TC), triglycerides (TGs), HDL-C, LDL-C, and urinary potassium and cortisol levels.

The prevalence of obesity, defined as a BMI ≥ 30 kg/m^2^ according to the Lancet Commission, was also assessed [48].

### 2.6. Dyslipidemia and Diabetes Diagnosis

According to the guidelines on dyslipidemia from the European Society of Cardiology [49], high total cholesterol (TC), high triglycerides (TGs), low HDL cholesterol (HDL-C), and high LDL cholesterol (LDL-C) were considered indicative of TC ≥ 200 mg/dL, TG ≥ 200 mg/dL, HDL-C < 50 mg/dL, and LDL-C ≥ 100 mg/dL, respectively. Dyslipidemia was diagnosed if any of the following conditions were met: elevated TC, elevated TG, low HDL-C, or high LDL-C.

Diabetes mellitus (DM) was diagnosed based on the WHO criteria, including a fasting plasma glucose ≥ 7.0 mmol/L, a 2 h post-glucose challenge plasma glucose (PG) ≥ 11.1 mmol/L, or current use of antidiabetic medication. The diagnostic threshold for HbA1c was set at ≥6.3% [50].

The presence of hypercortisolism was excluded in all patients by measuring 24 h urinary free cortisol levels.

### 2.7. Analysis Procedures

All biochemical parameters were measured using enzymatic methods on an autoanalyzer, except for plasma aldosterone concentrations (PACs), which were measured by chemiluminescence immunoassay using the commercially available Liaison Direct Renin (DIASORIN) kit. The intra- and inter-assay coefficients of variation (CVs) were 2.76% and 7.57%, respectively. Direct renin concentration (DRC) was also measured by chemiluminescence immunoassay using the same kit, with intra- and inter-assay CVs of 3.61% and 8.76%, respectively. Plasma cortisol concentrations were assessed using the Liaison (DIASORIN) kit. Samples with elevated cortisol levels were analyzed both as is and after serial dilution. The intra-assay and inter-assay CVs for cortisol were less than 10% and 15%, respectively.

### 2.8. Analytical Methods

Results are shown as averages ± standard deviations for variables with a normal distribution, medians with interquartile ranges (from the 25th to the 75th percentiles) for variables that are not normally distributed, and proportions for categorical variables. Differences between groups were assessed using Student’s *t*-test for variables following a normal distribution, and ANOVA for comparisons involving multiple groups, when applicable. The Mann–Whitney U test was employed for variables that did not follow a normal distribution, while the chi-square test was utilized for categorical data. Several logistic regression models were performed, controlling for the factors listed in the tables. The findings are presented as likelihood ratios (LRs) with 95% confidence intervals (CIs). *p*-values below 0.05 were considered statistically significant.

## 3. Results

The basic characteristics of the studied groups are shown in Table 1. We reported no differences in the age, sex, and duration of hypertension at the time of our investigation among the groups studied. Systolic blood pressure (SBP) and diastolic blood pressure (DBP) values were significantly higher in patients with PA compared to those with EH. In particular, both APA and BAH patients had significantly higher values (*p* = 0.001).

Serum potassium levels and the ARR in unilateral PA were greater than in bilateral forms or EH. Nevertheless, LDL-C and triglyceride levels were greater in bilateral PA than in unilateral forms. In contrast, serum LDL-C levels were significantly lower in unilateral PA compared to patients with EH. (Table 1).

**Figure 2:**
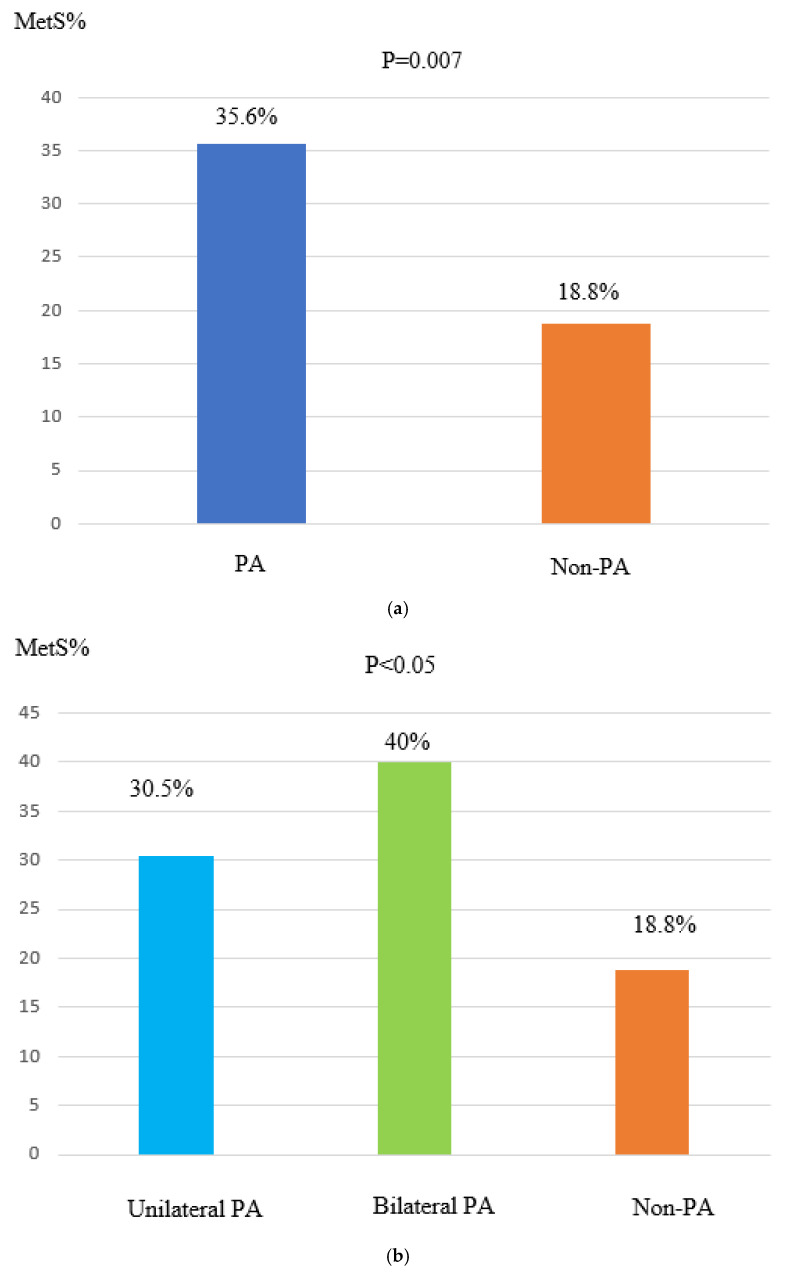

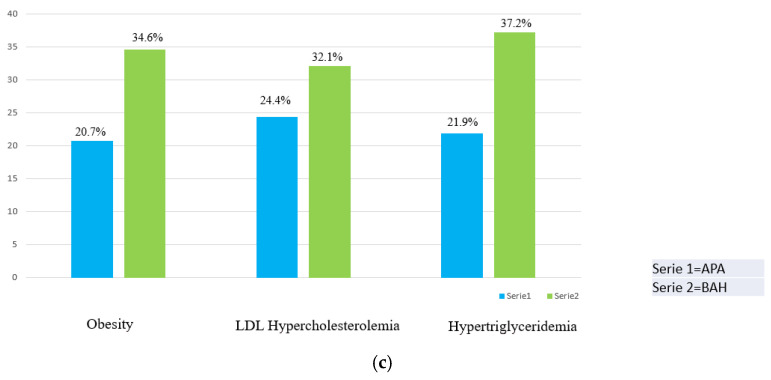
(**a**) The PA and non-PA groups did not show a significant difference in prevalence. (**b**) The incidence of MetS was higher in individuals with unilateral PA compared to those with bilateral PA and essential hypertension. (**c**) Greater prevalence of obesity, LDL hypercholesterolemia, and hypertriglyceridemia in bilateral PA compared to unilateral PA. Abbreviations: PA—patients with primary aldosteronism; Non-PA—patients without primary aldosteronism; MetS—metabolic syndrome; APA—aldosterone-producing adenoma; BAH—bilateral adrenal hyperplasia.

The incidence of metabolic syndrome (MetS) was higher in aldosteronism than in EH, with a statistically significant difference (*p* = 0.007) (Figure 2a).

This higher prevalence of MetS was particularly evident in individuals with bilateral PA, who exhibited a markedly greater prevalence of MetS than those with unilateral PA (41% vs. 30.5%; *p* = 0.001) (Figure 2b).

Obesity, hypercholesterolemia, and hypertriglyceridemia were also more prevalent in individuals with bilateral PA compared to those with unilateral PA (Figure 2c).

An analysis of the clinical and laboratory characteristics in PA patients with and without MetS or obesity revealed that individuals with MetS were characterized by an increased BMI, a greater incidence of BAH, and a greater male predominance compared to those without MetS. Conversely, obese patients tended to be younger and showed a higher prevalence of BAH than their non-obese counterparts (Table 2).

To investigate the relationship between BAH and MetS/obesity, a multivariate logistic model was used (Table 3).

A significant association was observed between BAH and MetS (OR: 2.398; 95% CI: 1.254–4.584; *p* = 0.0082). After controlling for age and sex, this association remained statistically significant. Furthermore, the multivariate logistic model demonstrated an independent link between BAH and obesity, even after accounting for age and sex.

The exact mechanisms responsible for the higher prevalence of MetS in BAH patients compared with APA are not clear and may involve several potential factors such as age, the duration of hypertension, or diabetes mellitus. No significant intergroup differences in age, the duration of hypertension, or diabetes mellitus were, however, found in our study. The median duration of hypertension was 4 years ± 1 in the PA group and 4 years ± 0.8 in the EH group, while there were 11 diabetics in the PA group and 9 in the EH group. Furthermore, all participants, including the controls, underwent screening for PA after the discontinuation of potentially interfering medications.

## 4. Discussion

Our analysis revealed a higher frequency of MetS in individuals with bilateral primary aldosteronism (PA), demonstrated by AVS, compared to those with unilateral PA. Nevertheless, individuals with APA exhibited an elevated ARR and reduced serum potassium levels compared to those with BAH. Additionally, after controlling for age and sex, the multivariate logistic model identified a distinct link between BAH, MetS, and obesity.

Although in several previous investigations the incidence of MetS is higher in individuals with PA than in those with essential hypertension [51,52,53], this remains a debated issue. Indeed, in the cross-sectional study by Matrozova et al., metabolic characteristics did not differ between individuals with PA and those with essential hypertension [54]. A number of conditions contribute to the debate, such as varying characterizations of MetS and the differing frequencies of individuals with BAH and APA, as shown in Table 4, which could lead to different incidences of MetS in individuals with PA.

A meta-analysis from last year compared 12 studies, concluding that patients with PA have a higher prevalence of MetS and vice versa [55], but it did not conduct a subtype subanalysis to distinguish the prevalence of MetS in patients with APA and BAH.

**Table 4:** Published series on PA and MetS.

	Number of Patients			Prevalence of MetS		
Published Series	Cases	Controls	BAH%	Cases	Controls	*p*
Fallo 2006 [52]	85 PA patients	381 EH patients	65.8%	41.1%	29.6%	<0.05
Somlòovà 2010 [39]	100 PA patients	90 EH patients	50%	39%	32.2%	not mentioned
Iacobellis 2010 [35]	75 PA patients	192 EH patients	50.7%	25.3%	20.8%	not mentioned
Ronconi 2010 [53]	89 PA patients	164 EH patients	68.5%	45%	30%	<0.05
Turchi 2014 [51]	66 PA patients	132 EH patients	60.8%	47%	32%	<0.05
Hanslik 2015 [56]	183 PA patients	183 controls, matched for age, sex and BP	not mentioned	56.8%	44.8%	=0.02
Monticone 2017 [57]	99 PA patients	1573 non-PA hypertensive patients	70.3%	45.4%	29.8%	<0.001
Zhang 2020 [40]	169 PA patients	169 non-PA hypertensive patients, matched for age and sex	49.7%	72.2%	65.7%	<0.05
Present study	160 PA patients	80 EH patients, matched for age and sex	48.8%	35.75%	18.8%	<0.05

Abbreviations: PA—patients with primary aldosteronism; MetS—metabolic syndrome; BAH—bilateral adrenal hyperplasia; non-PA—patients without primary aldosteronism; EH—patients with essential hypertension.

Indeed, the disparity in MetS incidence between the two forms of PA has only been directly investigated in a limited number of studies. According to a 2010 study [39], individuals with BAH had a significantly higher incidence of MetS than those with APA. The study also found that the metabolic characteristics of individuals with BAH were similar to those seen in patients with essential hypertension, but worse than those observed in individuals with APA. Similarly, a study by Zhang et al. [40] found that the incidence of MetS was significantly higher in BAH forms than in APA, aligning with our findings. These findings support our results. While the meta-analysis by Sun et al. does not report the disparity in MetS incidence between the two PA forms, our study confirms a significant difference in MetS, with patients with PA being at a disadvantage compared to those with EH. Furthermore, specifically, there is also a significant difference in MetS between APA and BAH, with the latter subtype being more affected, reinforcing the existing evidence. This should lead to a necessary increased attention during the diagnostic phases of subtype classification for these patients.

We also observed that obesity was more prevalent in patients with PA than in those with EH, and the difference was particularly related to the distinction between BAH and APA forms, with a statistically significant difference between EH and BAH in particular (*p* = 0.001).

Finally, it remained to be clarified that cholesterol and triglyceride levels appeared to be lower in patients with PA compared to those with EH, but no subtype subanalysis had been conducted. We performed a direct comparison between EH and APA and between EH and BAH, innovatively highlighting that there is no statistically significant difference in terms of hypertriglyceridemia between EH and APA and between EH and BAH (*p* = 0.136 and *p* = 0.515, respectively).

We also observed that LDL hypercholesterolemia and hypertriglyceridemia were more prevalent in BAH forms than in APA forms. This predominance may be a key determinant of the greater incidence of MetS and the increased cardiovascular risk associated with BAH forms.

APA and BAH have distinct etiologies, and somatic mutations in the KCNJ5 gene are found in 35–70% of APA cases, with a higher frequency in East Asian populations [58]. In contrast, increased autonomous aldosterone production in BAH is thought to be driven by mutations in CACNA1D and the associated enlargement or accumulation of the adrenal cortex [59]. However, the impact of these different pathogeneses on the onset of MetS in individuals with APA versus those with BAH remains unresolved. Moreover, our study found that BAH forms were separately linked to obesity.

Consistent with these findings, Ohno et al. [41] reported that individuals with bilateral PA have more obesity, although their PAC levels are lower than those of individuals with unilateral PA. This suggests that factors associated with obesity may play a role in the development of BAH. The authors of the aforementioned 2010 study [39] also observed a significantly higher BMI in patients with IHA compared to APA patients; another 2018 study [60] showed that individuals with bilateral forms of PA had significant associations between PAC levels and both visceral fat percentage and visceral fat area. Another 2015 study showed that obesity is linked to higher mineralocorticoid receptor (MR) expression in subcutaneous and visceral fat in both humans and mice. Specifically, the doxycycline-induced overexpression of MR in adipocytes for 12 weeks led to MetS-like alterations, such as glucose intolerance, elevated triglyceride levels, and increased cholesterol, without affecting circulating aldosterone or blood pressure levels [61]. Consequently, it is possible that the greater incidence of MetS in BAH may be linked to obesity.

Previous research has also highlighted the role of adipokines in stimulating aldosterone secretion from human adrenal cortex cells [62]. Insulin sensitivity and the glycometabolic profile are also regulated by substances secreted by adipose tissue, known as adipokines. These include, for example, adiponectin, leptin, and resistin [35,63,64]. Nevertheless, further studies are needed to clarify the interactions between MetS and BAH, taking into account the relationships between obesity, metabolic alterations, and BAH.

A 2017 study highlighted the metabolic alterations observed in patients with PA who also exhibit subclinical hypercortisolism (SH) [65]. A significant number of PA cases with SH are associated with solitary adenomas co-secreting both aldosterone and cortisol. Our group has reported one such aldosterone–cortisol adenoma that resulted in hypoadrenocorticalism following surgical removal [66]. In a 2019 study [67], it was observed that a high incidence of diabetes mellitus is found in individuals with PA, due to the co-presence of SH. In the same 2017 study cited above [65], various metabolic risk parameters, such as waist circumference, HDL cholesterol, and diastolic blood pressure, were also found to be associated with excess glucocorticoids. Abnormal adipose distribution and an increase in abdominal visceral fat could lead to chronic cortisol overload, contributing to significant metabolic disturbances and heightened cardiovascular risk [38]. However, to date, there is no evidence suggesting that BAH patients have a higher incidence of SH than APA patients. In the same 2019 study cited above [67], the authors state that in patients with BAH, the higher incidence of prediabetes is not explained by the potential co-occurrence of SH.

Within our research, while we excluded patients with Cushing’s syndrome, we omitted to execute the 1 mg dexamethasone suppression test in all individuals. Therefore, further research is needed to investigate the incidence of SH and the possible influence on MetS in patients with APA and BAH.

This work has some limitations that need to be acknowledged. As a cross-sectional study, it does not allow for the determination of the causal mechanisms correlating MetS with BAH. Furthermore, the prevalence of MetS may be higher because the study participants were recruited from a tertiary care center. Additional prospective studies will be required to address these issues.

## 5. Conclusions

In this study, individuals with BAH were more likely to have MetS than those with APA. Although patients with APA show a greater ARR and a more pronounced potassium decline, our findings suggest that APA and BAH may involve different processes contributing to the development of MetS. The relatively small cohort may limit the generalizability of the results. In addition, the retrospective analysis may introduce biases, such as incomplete data or selection bias. Finally, the study design limits causal inferences regarding the relationship between PA subtypes and MetS. Therefore, further studies are needed to confirm these results.

## Figures and Tables

**Table 1:** Clinical and laboratory features of studied EH, APA, and BAH.

	EH (n = 80)	APA (n = 82)	BAH (n = 78)	*p* Value APA vs. BAH	*p* Value EH vs. APA	*p* Value EH vs. BAH
Age (years)	55 ± 0.66	49 ± 0.70	51 ± 0.69	0.126	0.342	0.774
Sex (male), n (%)	38 (47.5%)	37 (45.1%)	38 (48.7%)	0.385	0.874	0.878
SBP (mmHg)	139 ± 0.42	180 ± 0.36	180 ± 0.36	0.37	0.001	0.001
DBP (mmHg)	90 ± 0.52	105 ± 0.48	110 ± 0.47	0.07	0.001	0.001
K+ (mmol/L)	4.1 ± 2.45	3.8 ± 2.83	4 ± 2.45	0.006	0.006	0.007
PAC (ng/dL)	99 ± 0.49	22.3 ± 0.33	21.8 ± 0.33	0.203	0.001	0.001
ARR	11 ± 1.48	122 ± 0.44	65.8 ± 0.60	0.002	0.001	0.001
BMI (kg/m^2^)	25 ± 0.98	28.6 ± 0.92	27.8 ± 0.94	0.201	0.099	0.8
Tryglicerides (mg/dL)	98 ± 0.49	96 ± 0.50	104 ± 0.48	0.046	0.136	0.515
HDL-C (mg/dL)	51.5 ± 0.69	48 ± 0.70	48 ± 0.70	0.282	0.627	0.219
LDL-C (mg/dL)	117 ± 0.45	95.6 ± 0.50	112 ± 0.46	0.046	0.041	0.184
TC (mg/dL)	187 ± 0.36	173 ± 0.37	184 ± 0.36	0.07	0.328	0.381
glycemia (mg/dL)	91 ± 0.51	89 ± 0.51	91 ± 0.51	0.371	0.841	0.249
DM, n (%)	9 (11.3%)	6 (7.3%)	5 (6.4%)	0.118	0.388	0.284
Obesity, n (%)	20 (25%)	17 (20.7%)	27 (34.6%)	0.001	0.518	0.046
MetS, n (%)	15 (18.8%)	25 (30.5%)	32 (41%)	0.001	0.083	0.002

Abbreviations: EH—essential hypertension; APA—aldosterone-producing adenoma; BAH—bilateral adrenal hyperplasia; SBP—systolic blood pressure; DBP—diastolic blood pressure; K^+^—kalemia; PAC—plasma aldosterone concentration; ARR—aldosterone–renin ratio; BMI—body mass index; HDL-C—HDL cholesterolemia; LDL-C—LDL cholesterolemia; TC—total cholesterolemia; DM—diabetes mellitus; MetS—metabolic syndrome.

**Table 2:** Clinical and laboratory features of patients with PA with or without MetS/obesity.

Subtype (BAH), n (%)	Without MetS (n = 46)	With MetS (n = 32)	*p*	Without Obesity (n = 51)	With Obesity (n = 27)	*p*
Age (years)	50 ± 0.69	50 ± 0.69	0.435	50 ± 0.69	42 ± 0.76	0.046
Sex (males), n (%)	33 (72%)	16 (50%)	0.008	26 (51%)	17 (63%)	0.093
BMI (kg/m^2^)	25.3 ± 0.98	29.65 ± 0.90	0.036	25.45 ± 0.98	31.3 ± 0.88	0.952
K^+^ (mmol/L)	4 ± 2.45	4 ± 2.45	0.62	4 ± 2.45	4 ± 2.45	0.465
PAC (ng/dL)	186 ± 0.36	199 ± 0.35	0.715	197 ± 0.35	168.5 ± 0.38	0.622
ARR	82.7 ± 0.54	57.5 ± 0.65	0.822	72 ± 0.58	53.75 ± 0.67	0.301

Abbreviations: PA—patients with primary aldosteronism; MetS—metabolic syndrome; BAH—bilateral adrenal hyperplasia; BMI—body mass index; K^+^—kalemia; PAC—plasma aldosterone concentration; ARR—aldosterone–renin ratio.

**Table 3:** Association between BAH and MetS/obesity.

	MetS		Obesity	
Adjusted variables	OR (95% CI)	*p* Value	OR (95% CI)	*p* Value
Model 1 *	2.398 (1.2544–4.5844)	0.008	3.667 (1.8745–7.1721)	0.001
Model 1 * + age	2.397 (1.2544–4.5844)	0.008	3.688 (1.8739–7.1825)	0.003
Model 1 * + sex	2.410 (1.2662–4.5990)	0.013	3.544 (1.8673–7.1514)	0.009

* Model 1: unadjusted model. Abbreviations: CI—confidence interval; MetS—metabolic syndrome; OR—odds ratio; BAH—bilateral adrenal hyperplasia.

## Data Availability

The raw data supporting the conclusions of this article will be made available by the authors on request.
